# Synthesis, radiolabeling, and evaluation of a (4-quinolinoyl)glycyl-2-cyanopyrrolidine analogue for fibroblast activation protein (FAP) PET imaging

**DOI:** 10.3389/fbioe.2023.1167329

**Published:** 2023-03-28

**Authors:** Ni Zhang, Fei Pan, Lili Pan, Wei Diao, Feijing Su, Rui Huang, Bo Yang, Yunchun Li, Zhongzhi Qi, Wenjie Zhang, Xiaoai Wu

**Affiliations:** ^1^ Department of Nuclear Medicine, Laboratory of Clinical Nuclear Medicine, National Clinical Research Center for Geriatrics, West China Hospital, Sichuan University, Chengdu, Sichuan, China; ^2^ Department of Psychiatry, West China Hospital of Sichuan University, Chengdu, China; ^3^ Core Facilities of West China Hospital, Sichuan University, Chengdu, China; ^4^ Department of Neurology, Sichuan Academy of Medical Science and Sichuan Provincial People’s Hospital, Chengdu, China; ^5^ Department of Pharmacy, The Seventh People’s Hospital of Chengdu, Chengdu, China; ^6^ Department of Pharmacy, Nanchong Central Hospital, The Second Clinical Medical College, North Sichuan Medical College, Nanchong, China

**Keywords:** FAP, 18F-labeled FAPI, biodistribution, PET/CT imaging, blood–brain barrier

## Abstract

Fibroblast activation protein (FAP) is regarded as a promising target for the diagnosis and treatment of tumors as it was overexpressed in cancer-associated fibroblasts. FAP inhibitors bearing a quinoline scaffold have been proven to show high affinity against FAP *in vitro* and *in vivo*, and the scaffold has been radio-labeled for the imaging and treatment of FAP-positive tumors. However, currently available FAP imaging agents both contain chelator groups to enable radio-metal labeling, making those tracers more hydrophilic and not suitable for the imaging of lesions in the brain. Herein, we report the synthesis, radio-labeling, and evaluation of a ^18^F-labeled quinoline analogue ([^18^F]**3**) as a potential FAP-targeted PET tracer, which holds the potential to be blood–brain barrier-permeable. [^18^F]**3** was obtained by one-step radio-synthesis *via* a copper-mediated S_N_A_R_ reaction from a corresponding boronic ester precursor. [^18^F]**3** showed moderate lipophilicity with a log *D*_*7.4*_ value of 1.11. In cell experiments, [^18^F]**3** showed selective accumulation in A549-FAP and U87 cell lines and can be effectively blocked by the pre-treatment of a cold reference standard. Biodistribution studies indicated that [^18^F]**3** was mainly excreted by hepatic clearance and urinary excretion, and it may be due to its moderate lipophilicity. *In vivo* PET imaging studies indicated [^18^F]**3** showed selective accumulation in FAP-positive tumors, and specific binding was confirmed by blocking studies. However, low brain uptake was observed in biodistribution and PET imaging studies. Although our preliminary data indicated that [^18^F]**3** holds the potential to be developed as a blood–brain barrier penetrable FAP-targeted PET tracer, its low brain uptake limits its application in the detection of brain lesions. Herein, we report the synthesis and evaluation of [^18^F]**3** as a novel small-molecule FAPI-targeted PET tracer, and our results suggest further structural optimizations would be needed to develop a BBB-permeable PET tracer with this scaffold.

## 1 Introduction

As a type II transmembrane serine protease, fibroblast activation protein (FAP) belongs to the dipeptidyl peptidase (DPP) 4 protein family and is highly expressed in the cancer-associated fibroblasts in about 90% of normal human epithelial tumors (Garin-Chesa et al., 1990; Scanlan et al., 1994; Hamson et al., 2014; Gascard and Tlsty, 2016). The expression of FAP was also found to be associated with poor prognosis in a variety of malignant tumors, such as ovarian cancer, pancreatic cancer, colon cancer, and hepatocellular carcinoma (Henry et al., 2007; Cohen et al., 2008; Ju et al., 2009; Zhang et al., 2011). Although the positive expression of FAP (especially FAP-α) cannot be confirmed in several tumor cell lines with Western blot or QT-PCR techniques, it can be readily detected in tumor stroma (Lee et al., 2005). Therefore, FAP is regarded as a promising target for the diagnosis and treatment of tumors.

**FIGURE 1:**
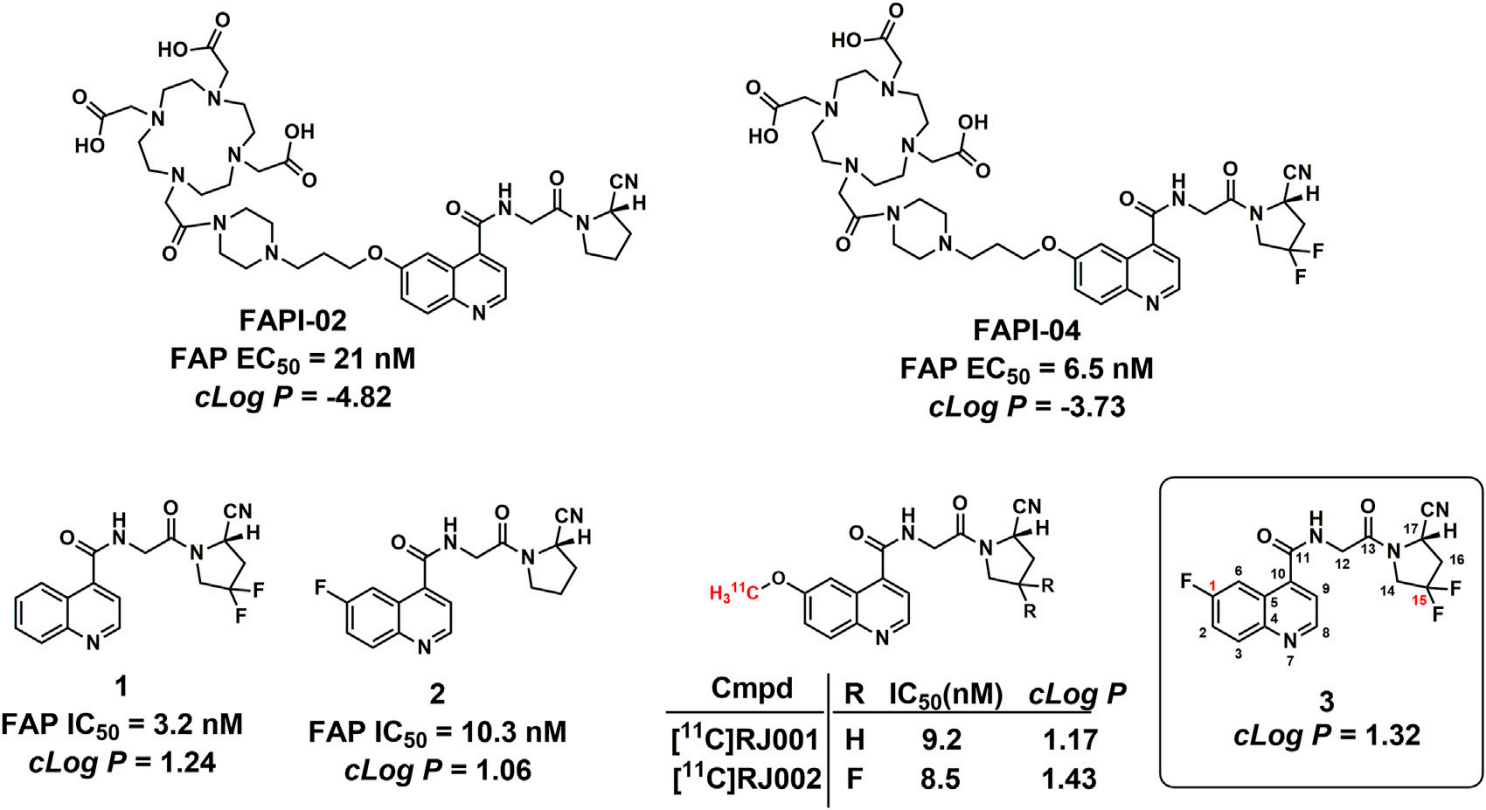
Structures of the representative FAPI PET tracers and compound 1. IC_50_ values for FAP-02, FAP-04, and compounds **1** and **2** were obtained from published literature reports (Jansen et al., 2013; Jansen et al., 2014; Lindner et al., 2018).

To date, multiple approaches have been developed for tumor treatment by targeting FAP, such as anti-FAP antibodies (Welt et al., 1994; Hofheinz et al., 2003; Scott et al., 2003), vaccines (Lee et al., 2005; Loeffler et al., 2006), immunoconjugates (Ostermann et al., 2008), CAR-T cells, and FAP inhibitors (Wang et al., 2014; Lo et al., 2015; Teichgräber et al., 2015; Lindner et al., 2018). In addition, another approach is the radio-labeling of FAP-targeting molecules, with alpha-emitting nuclides, beta-emitting nuclides, or positron-emitting radionuclides, to achieve both *in vivo* imaging and treatment of FAP-positive tumors. With the development of highly potent quinoline-based inhibitors for FAP in recent years, the radionuclide-based approach is more focused on small-molecule FAP inhibitors. However, in order to achieve the integration of imaging and therapy, chelating groups for radioactive metals such as DOTA were introduced to the scaffold of the quinoline-based analogues to enable radio-metal radiolabeling (most therapeutic radionuclides are metal elements), which made these ligands more hydrophilic and not suitable for the imaging of lesions in the urinary system (high physiological accumulation caused high background radioactive signals), such as FAPI-01(^125^I-labeled), [^68^Ga/^177^Lu]FAPI-02, and [^68^Ga, ^90^Y/^177^Lu]FAPI-04 (Figure 1) (Lindner et al., 2019). In addition, as FAP expression was also found in glioblastoma tissues in the brain (Ebert et al., 2020), the development of blood–brain barrier-permeable FAP-targeted PET tracer is highly valuable for the detection of brain tumors and brain metastases (Zhang et al., 2021; Wei et al., 2022), but the hydrophilic chelating groups such as DOTA and NOTA made the conjugates less possible to penetrate the blood–brain barrier and reach the target in the brain. (Ballal et al., 2021; Giesel et al., 2021; Yang et al., 2021)

At present, small-molecular FAP inhibitors bearing a quinoline-4-carboxamide scaffold showed high accumulation in FAP-positive cells, tumors, and patients. Therefore, quinoline-4-carboxamide analogues are promising lead compounds to develop BBB-permeable FAP PET tracers. According to the structure–activity relationship (SAR) revealed by FAPI-02, FAPI-04, compound **1,** and compound **2** (Figure 1), the introduction of two fluorine atoms at the C-15 position greatly increased enzymatic inhibitory activity against FAP (FAPI-02 and FAPI-04), and the substituents with different sizes at the C-1 position have minimal impact on the bioactivities against FAP (FAPI-02 and compound **2**) (Jansen et al., 2013; Jansen et al., 2014; Lindner et al., 2018). Liu et al. reported the *in vivo* evaluations of ^11^C-radiolabeled quinoline-4-carboxamide analogues, ^11^C-RJ001 and [^11^C]RJ002 (Figure 1), and found that [^11^C]RJ002 showed higher tumor accumulation than [^11^C]RJ001 (Wang et al., 2022).

With a longer physical half-life, ^18^F (109.8 min) is more convenient than ^11^C (20.4 min) to produce PET tracers for clinical use. In this study, we performed structural modification on a quinoline-4-carboxamide scaffold with previous experience, and one fluorine atom was introduced to the C1 position of the quinoline ring to prepare the more potent inhibitor with higher lipophilicity (compound **3**, Figure 1), and the corresponding ^18^F-radiolabeled compound was also successfully prepared and evaluated *in vitro* and *in vivo*. With the removal of the chelator group, compound **3** showed higher lipophilicity with a *ClogP* value of 1.32 than those of FAPI-02 (-4.82) and FAPI-04 (-3.73) and also similar to that of the recently reported FAP-targeted small molecule [^11^C]RJ002 (1.43), which showed more significant brain uptake than [^11^C]RJ001 (1.17) and [^68^Ga]FAPI-04 (-3.73) in mouse models. The *ClogP* values were calculated based on a compound physicochemical property evaluation platform (Dong et al., 2021; Xiong et al., 2021).

Herein, we report the synthesis, radio-labeling, and evaluation of a ^18^F-labeled (4-quinolinoyl)glycyl-2-cyanopyrrolidine analogue as a potential FAP-targeted PET tracer. However, further optimization is needed for this (4-quinolinoyl)glycyl-2-cyanopyrrolidine scaffold to be developed as a PET tracer for the detection of FAP in the brain.

## 2 Results

### 2.1 Compound synthesis

**SCHEME 1:**
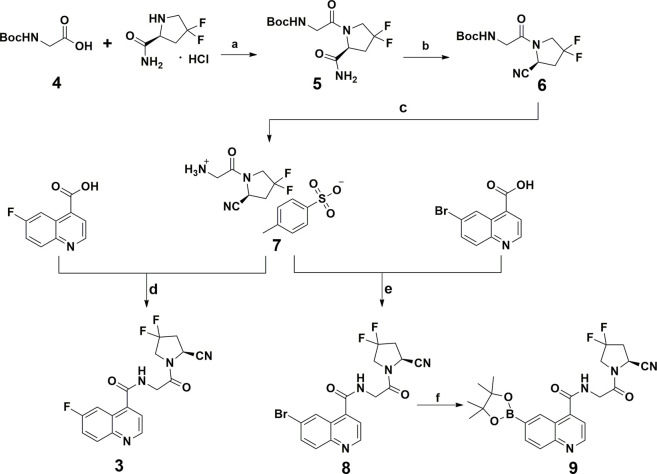
Chemical synthesis route of the reference standard and radio-labeling precursor. Reagents and conditions: **(A)** EDCI, HOBT, DIPEA, DMF, rt; **(B)** trifluoroacetic anhydride, pyridine, THF, 0°C; **(C)** p-Toluenesulfonic acid, CH_3_CN, rt; **(D, E)** EDCI, HOBT, DIPEA, DMF, rt; **(F)** B_2_Pin_2_, KOAc, PdCl_2_(dppf), DMF, 80°C, 18 h.

The synthetic procedure for all compounds in this study is presented in Scheme 1. All compounds were readily obtained and characterized by NMR and HRMS spectra. Detailed information can be found in Section 5.

### 2.2 Radiosynthesis of [^18^F]3

**FIGURE 2:**
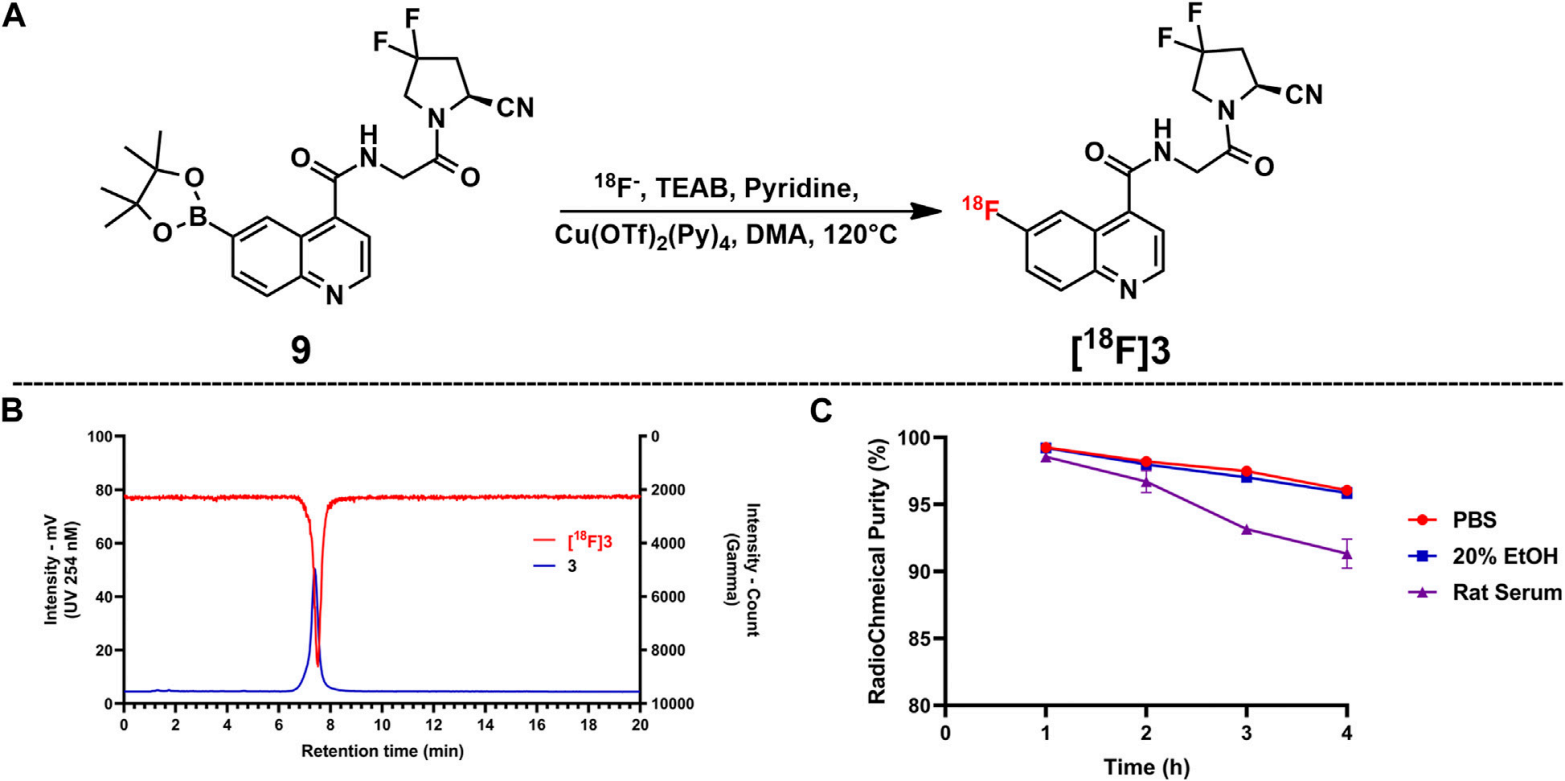
Copper-mediated S_N_A_R_ radiofluorination *via* the boronic ester precursor and *in vitro* tests for RCY and stability. **(A)** Radiolabeling reaction. **(B)** Co-injection of the reference standard (compound **3**) and [^18^F]**3**; compound **3** showed a retention time of 7.40 min and that of [^18^F]**3** was 7.48 min. **(C)** Radiochemical purities for all samples in the stability test; bars indicate SD.

[^18^F]**3** was produced by classic copper-mediated nucleophilic fluorination with the corresponding boronic ester precursor (compound **9**) (Tredwell et al., 2014), as shown in Figure 2. The total synthesis time for [^18^F]**3** is about 70 min from the delivery of the radionuclide to the end of formulation, and the radiochemical yield is 43.4% ± 8.5% (n = 4). According to the co-injection of the final product and the cold reference standard (compound **3**) into the radioanalytical HPLC, [^18^F]**3** showed a retention time of 7.48 min (gamma signal) and that of the reference standard was 7.40 min (UV 254 nM signal), indicating the consistency of the final product and standard. Based on the activity of the injected final product and integration of the product peak from the ultraviolet signal (254 nm) on the analytical HPLC system from another injection with [^18^F]**3**, molar activity of [^18^F]**3** was calculated with the standard curves generated from compound **1** at the same condition. The radio-chemical purity of [^18^F]**3** was greater than 99%, and molar activity was 287.5 ± 60.4 GBq/µmol after formulation.

### 2.3 *In vitro* physicochemical properties

The log *D*_*7.4*_ value of [^18^F]**3** was 1.11 ± 0.04, which indicates that [^18^F]**3** possesses moderate lipophilicity for BBB and other passive membrane penetration. In addition, with higher lipophilicity than that of DOTA or NOTA conjugates, the accumulation of [^18^F]**3** in the urinary system may be lower than that of a previously reported chelator containing FAP PET tracer, implying a higher tumor-to-background ratio in the urinary system.

The stability profile of [^18^F]**3** in PBS saline, 20% EtOH solution, and rat serum is presented in Figure 2. According to the stability tests, [^18^F]**3** in the rat serum is lower than that of PBS and EtOH solution, but over 90% intact [^18^F]**3** in all tested solutions was observed after incubation for 4 h at 37°C, indicating the high *in vitro* stability of this compound.

### 2.4 Cellular uptake studies

**FIGURE 3:**
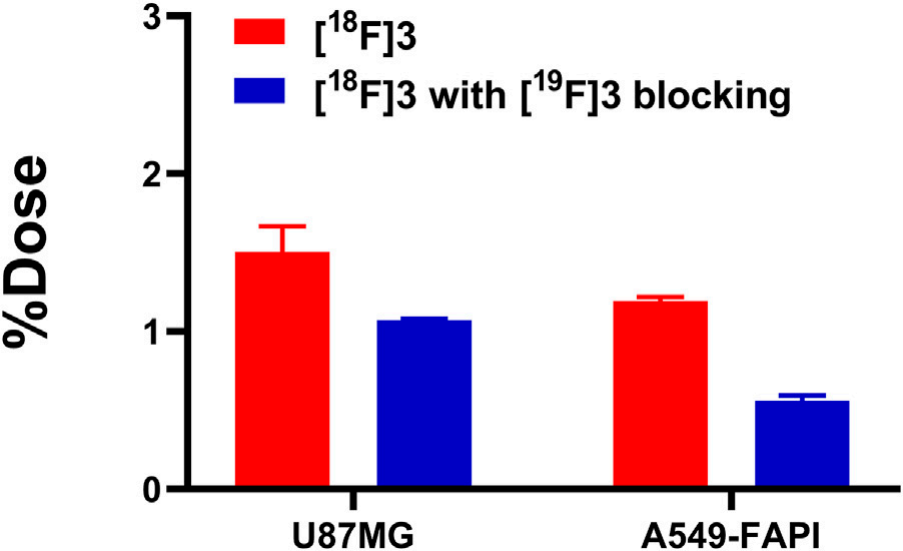
Cellular uptakes of [^18^F]**3** in U87MG and A549-FAPI cells.

As shown in Figure 3, data for the cellular uptake studies are presented as a percentage of the applied dose (%Dose). [^18^F]**3** showed relatively higher accumulation in U87 cells and A549-FAPI cells after 1 h of incubation, with uptake ratios of 1.50% ± 0.13% and 1.07% ± 0.01%, respectively. In blocking groups, accumulation can be effectively blocked by the pre-treatment of compound **3** (with the final concentration of 10 µM) and was reduced to 1.19% ± 0.02% and 0.56% ± 0.03% in these cells (reduced to 79.38% and 52.34% of the corresponding vehicle groups), indicating the selective binding of [^18^F]**3** to these cells.

### 2.5 Biodistribution studies

**FIGURE 4:**
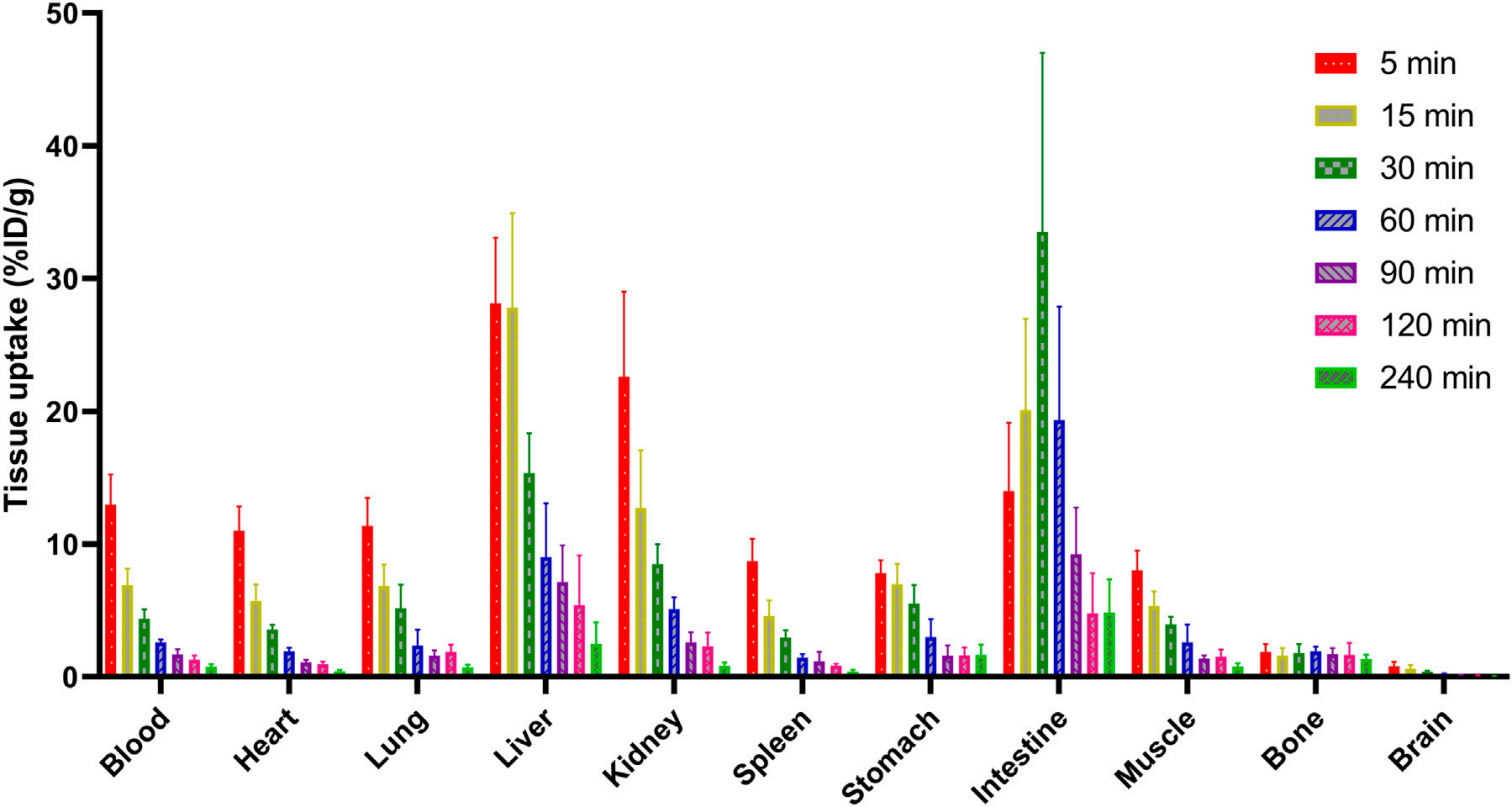
Biodistribution profile of [^18^F]**3** in normal Kunming mice.

The biodistribution analysis of [^18^F]**3** was performed in normal Kunming mice, as shown in Figure 4; Table 1. All data were presented as the percentage of injected dose per gram of organ/tissue, %ID/g. As shown in Figure 4 and Table 1, radioactivity was fast cleared from the blood pool, and only 2.61 %ID/g remained in the blood 1 h post-injection (p.i.). The primary mode of clearance for [^18^F]**3** was liver metabolism, with a tracer uptake of up to 28 %ID/g at 5 min p.i. in the liver, and the radioactivity gradually transferred to the lower gastrointestinal tract, with activity peaking at 30 min p.i. (33.51 %ID/g). [^18^F]**3** was also excreted from the urinary system, and the initial high kidney uptake was observed (22.59 %ID/g) and was fast cleared within 120 min. However, although [^18^F]**3** showed a suitable log *D*_*7.4*_ value of 1.11, low radioactivity was observed in the brain (lower than 1 %ID/g), suggesting a low BBB penetration ability of this tracer and not being suitable for the detection of primary brain tumors or metastasis. [^18^F]**3** showed high *in vivo* stability, and low activity was observed in the bone (lower than 2 %ID/g), indicating an absence of ^18^F-fluoride from metabolism. In addition, all other major organs and tissues showed low tracer uptake, and no obvious activity accumulation was observed during the study.

**TABLE 1:** Biodistribution data on [^18^F]3 in normal Kunming mice.

Tissue	Post-injection time (min)					
	5	15	30	60	120	240
Blood	12.96 ± 2.03	6.90 ± 1.13	4.35 ± 0.66	2.61 ± 0.18	1.29 ± 0.28	0.75 ± 0.19
Heart	11.00 ± 1.64	5.71 ± 1.12	3.53 ± 0.33	1.91 ± 0.24	0.93 ± 0.20	0.39 ± 0.11
Lungs	11.36 ± 1.89	6.84 ± 1.43	5.15 ± 1.59	2.35 ± 1.07	1.87 ± 0.49	0.69 ± 0.19
Liver	28.13 ± 4.42	27.82 ± 6.35	15.34 ± 2.67	9.03 ± 3.62	5.39 ± 3.36	2.47 ± 1.47
Kidney	22.59 ± 5.74	12.72 ± 3.88	8.48 ± 1.34	5.07 ± 0.81	2.27 ± 0.93	0.82 ± 0.23
Spleen	8.71 ± 1.50	4.56 ± 1.09	2.95 ± 0.49	1.46 ± 0.24	0.85 ± 0.13	0.38 ± 0.13
Stomach	7.82 ± 0.86	6.98 ± 1.36	5.50 ± 1.26	2.98 ± 1.22	1.63 ± 0.51	1.64 ± 0.69
Intestine	13.99 ± 4.60	20.10 ± 6.14	33.51 ± 12.05	19.33 ± 7.66	4.75 ± 2.73	4.84 ± 2.23
Muscle	8.02 ± 1.34	5.32 ± 1.02	3.92 ± 0.55	2.60 ± 1.20	1.51 ± 0.48	0.76 ± 0.23
Bone	1.86 ± 0.54	1.59 ± 0.51	1.80 ± 0.60	1.91 ± 0.33	1.65 ± 0.81	1.33 ± 0.30
Brain	0.80 ± 0.29	0.61 ± 0.25	0.35 ± 0.10	0.20 ± 0.04	0.08 ± 0.02	0.04 ± 0.02

### 2.6 PET imaging

**FIGURE 5:**
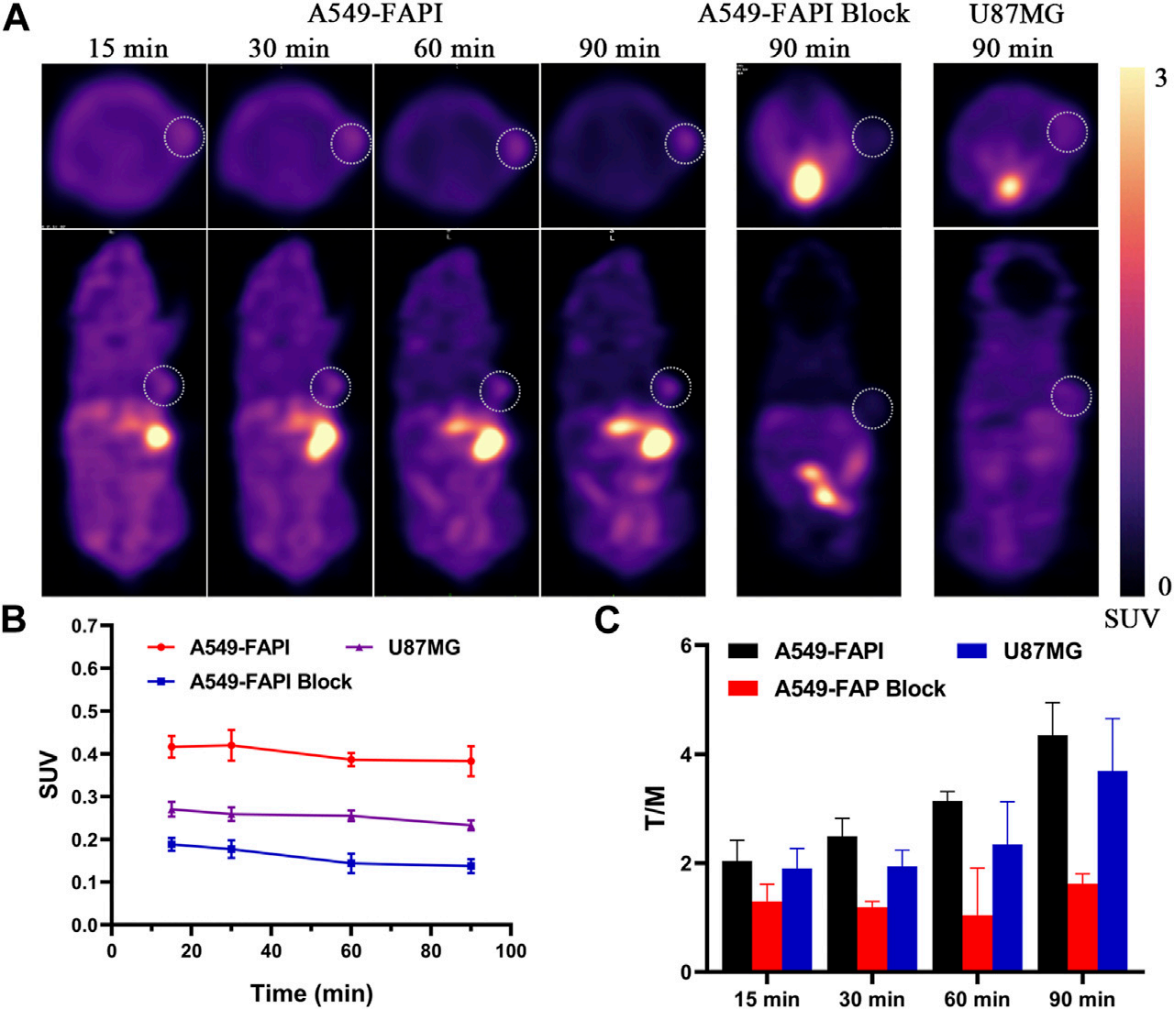
*In vivo* micro-PET imaging of FAP in tumors. **(A)** Static micro-PET images at all time points. **(B)** Tumor SUVs of [^18^F]**3** in all subjects; white cycles indicate the tumor lesions, and the scale bars show SUV values. **(C)** Tumor/muscle ratios calculated from PET images.

As shown in Figure 5, nude mice bearing A549-FAPI and U87MG xenograft tumors were scanned using an IRIS Micro-PET/CT system with [^18^F]**3.** Representative static images were obtained after a tail vein injection of [^18^F]**3** within 90 min. The distribution properties of this tracer in the micro-PET study were well agreed with the results in the biodistribution study, and a major metabolic pathway in the liver was observed in all tested subjects. The SUV values in the liver were about 5.0 at 15 min p.i. and gradually decreased to 2.0 at the end of the scan. The initial SUV values in the kidney were approximately 5.0 and also decreased to 3.0 at 90 min p.i., and the radioactivity gradually accumulated in the bladder with SUV values greater than 8.0 at the end of the scan. In addition, low activities were observed in the brain (SUV values less than 0.3) and therefore confirmed the low BBB penetration of this tracer. In A549-FAPI xenografted tumors, stable retention of radioactivity was observed with SUV values greater than 0.4 within 90 min, and this uptake can be effectively blocked by the pre-injection of the cold reference standard (compound **3**, 2 mg/kg) with significant inhibition of tumor SUV values (lower than 0.2, over 50% inhibition), indicating the selectivity of [^18^F]**3** to FAP. In addition, tumor SUV values were also greater than 0.22 in U87MG xenografted tumors within 90 min. With the extension of time intervals between the imaging and tracer injection, tracer accumulation in muscle, liver, and other major organs gradually decreased and tumor lesions were more clearly visualized. Tumor-to-muscle (T/M) ratios of 4.34 and 3.68 were obtained in A549-FAPI and U87MG tumors at 90 min p.i., respectively. In the blocking group, the T/M ratios were less than 2.0 in this study.

## 3 Discussion

FAP expression was proven to be associated with a poor prognosis in a kind of malignant tumor, and its overexpression in cancer-associated fibroblasts made it an ideal target for tumor imaging and therapy. In previous studies, a series of quinolone scaffold-based small molecules were developed as potential inhibitors targeting FAP, and these inhibitors were also labeled with a variety of diagnostic radionuclides for *in vivo* imaging of FAPI. In addition, highly potent FAP inhibitors were also labeled with therapeutic radionuclides to achieve the integration of diagnosis and therapy, i.e., theragnostics. These radiotracers have achieved remarkable performance in the diagnosis of FAP-positive tumors and have been used in clinical practice. However, currently available FAPI radiotracers contain highly water-soluble chelator groups to enable radiolabeling, such as DOTA and NOTA, making these tracers highly hydrophilic and not suitable for imaging brain lesions. Our research interest in this scaffold lies in the high inhibitory efficacy against FAP, and previous SAR studies of this scaffold revealed a fluoride-containing molecule, compound **3**, which showed nanomolar activity and can be labeled with ^18^F *via* direct radiofluorination. In addition, without the highly water-soluble chelator group, this compound holds the potential to be developed as a promising FAP PET tracer with BBB penetration.

[^18^F]**3** was successfully prepared, and it showed high radiochemical purity and molar activity after the final formulation. According to the *in vitro* tests for physicochemical parameters, [^18^F]**3** showed high *in vitro* stability and moderate lipophilicity with a log *D*_*7.4*_ value of 1.11, which is moderate for BBB penetration.

Then, a cellular uptake assay was performed to evaluate the selective binding of this tracer to FAP in A549-FAP and U87 cell lines, which were both FAP-positive cell lines. As a result, both cells showed selective accumulation of radioactivities, and this accumulation can be blocked by the pre-treatment of 10 µM of the reference standard (compound **3**), indicating the specific binding of [^18^F]**3** to FAP. In agreement with the moderate lipophilicity of [^18^F]**3,** the biodistribution profile indicated this tracer was mainly metabolized in the liver and excreted through the hepatobiliary pathway. However, a considerable percentage of radioactivity was also excreted through the urinary system. [^18^F]**3** showed distribution in the muscles, stomach, heart, and lung, which may result from the lipophilicity of this compound. In addition, low radioactivity in the bone was observed, indicating the absence of ^18^F-fluoride from defluorination and the *in vivo* stability of this compound.

With physicochemical parameters and biodistribution profiles in hand, [^18^F]**3** was further evaluated in PET imaging studies with nude mice bearing A549-FAPI and U87MG xenografted tumors. PET images in all subjects well agreed with the biodistribution data, and significant accumulation of radioactivity in the liver, digestive tract, kidney, and bladder was observed. As expected, [^18^F]**3** also showed accumulation in FAP-positive tumors, and T/M ratios of 4.34 and 3.67 peaked at 1.5 h p.i. for A549-FAPI and U87MG tumors, respectively. In addition, tumor uptake can be effectively blocked by the pre-injection of the reference standard in A549-FAPI tumors, suggesting the specific binding of [^18^F]**3** to FAP *in vivo*. However, relatively low brain uptake was observed in biodistribution and PET imaging studies, indicating [^18^F]**3** is not suitable for the detection of brain lesions with FAP expression. We think that is because the log *D*_*7.4*_ values and other physicochemical properties of [^18^F]**3** are not suitable for BBB penetration**,** and since [^11^C]RJ002 showed considerable brain uptake with higher C*logP* values than [^18^F]**3**, a higher log *D*_*7.4*_ may lead to higher brain uptake for this scaffold. Furthermore, to develop BBB-permeable FAP-targeted PET tracers, optimized physicochemical parameters for CNS small-molecule PET tracers (Zhang et al., 2013), i.e., MW (molecular weight), *ClogP* (calculated partition coefficient), Clog *D*_*7.4*_ (calculated distribution coefficient at pH = 7.4), TPSA (topological polar surface area), HBD (number of hydrogen bond donors), and pKa (acid dissociation constant of the most basic center), may also be used to optimize the quinolone scaffold with potent inhibitory activity against FAP in the brain.

## 4 Conclusion

In summary, we report the preparation and evaluation of a fluorine-18-labeled FAP inhibitor ([^18^F]**3**). *In vitro* studies indicated this radiotracer to possess promising properties as a BBB-permeable FAP-targeted PET tracer. However, *in vivo* studies showed low brain uptake. [^18^F]**3** showed selective accumulation in the cellular study and PET imaging study with FAP-positive cell lines and tumor models, and the blocking studies confirmed specific binding to FAP. [^18^F]**3** also showed slightly higher accumulation in non-targeted tissues and organs with higher lipophilicity than other chelators containing FAPI PET tracers. In a word, we provided a novel small-molecule FAPI PET tracer without chelator groups; [^18^F]**3** showed efficacy in detecting FAP-positive tumors *in vivo*, but further investigations were needed to develop BBB-permeable FAP-targeted PET tracers with this scaffold.

## 5 Materials and methods

### 5.1 General information

All reagents, compounds, and solvents were obtained from commercial suppliers and used without further processing unless indicated. Thin layer chromatography was used to monitor organic reactions, and flash column chromatography was used to purify the products. ^1^H NMR and ^13^C NMR spectra were performed on a spectrometer (Bruker AV-400, 400 MHz) in the Analysis and Testing Center of Sichuan University, and coupling constants are indicated as J with Hz (hertz); multiplicities are expressed as s; and d, t, q, and m for singlet, doublet, triplet, quartet, and multiplet, respectively. High-resolution mass spectrometry (HRMS) was carried out in a Q-TOF Premier mass spectrometer (Waters) in the State Key Laboratory of Biotherapy, Sichuan University.

All protocols related to animals in this investigation were performed with the approval of the Animal Management Committee of Sichuan University.

### 5.2 Compound synthesis

All compounds included in this study were produced according to the reported literature reports with minor modifications, as presented in Scheme 1. (Poplawski et al., 2013) In brief, compound **5** was obtained *via* amide condensation from the starting material boc-protected 2-aminoacetic acid (compound **4**) and (*S*)-4,4-difluoropyrrolidine-2-carboxamide and was converted to the cyano-containing compound (compound **6**) under dehydration conditions. Deprotection of compound **6** affords corresponding quaternary ammonium salt (compound **7**) and was used to prepare the reference standard (compound **3**) and key intermediate (compound **8**) for the ^18^F-radiolabeling precursor (compound **9**) *via* 6-fluoroquinoline-4-carboxylic acid and 6-bromoquinoline-4-carboxylic acid, respectively. Detailed information and characterization data can be found in the supplemental materials.

### 5.3 Radio-labeling of [^18^F]3

Classic copper-mediated nucleophilic fluorination was used in the radiolabeling of [^18^F]**3**, as shown in Figure 2 (Tredwell et al., 2014). Briefly, ^18^F-fluoride was produced (HM-10 cyclotron, Sumitomo Heavy Industries, Ltd., Japan), transferred, and trapped on a Sep-Pak Light QMA cartridge (Waters) installed in the automated module (PET-MF-2V-IT-1, Beijing PET Technology, China). Eluted by the tetraethylammonium bromide (TEAB) solution (2 mg of TEAB in 0.7 mL of acetonitrile and 0.3 mL of water), the radioactivity in the QMA cartridge was transferred to the reaction tube. The solvent was then removed by azeotropic drying at 110°C under a gentle stream of nitrogen three times, and after cooling to room temperature, the reaction mixture containing 2 mg of compound **9**, 4 mg of copper catalyst, 20 µL of pyridine, and 1 mL of DMA was added to the reaction tube and was heated at 120°C for 20 min. After dilution with 5 mL of water, the reaction mixture was injected into the online semi-preparative HPLC system for purification. The HPLC system was equipped with an Alltech Chrom BDS 10u column (250 mm * 10 mm) and eluted with a mobile phase of acetonitrile and ultra-pure water containing 0.1% HCOOH (50/50, V/V) at a flow rate of 4 mL/min. The product peak displayed a retention time of 15 min and was collected, diluted with 100 mL of water, and then passed through a pre-activated (with ethanol) Waters C18 light cartridge. The final product was washed off from the cartridge by the elution of 0.5 mL of ethanol and was formulated with 9.5 mL of sterilized water for further evaluation. The identification of [^18^F]**3** was performed by the co-injection of the final product and the cold reference standard (compound **3**) on an analytical radio-HPLC system, which was equipped with a Phenomenex Gemini 5 µm C18 (2) column (250 mm * 4.6 mm) and eluted by acetonitrile and pH 8.0 PBS buffer (40/60, V/V) at a flow rate of 1.5 mL/min.

### 5.4 *In vitro* physicochemical properties

The lipid/water partition coefficient of [^18^F]**3** was tested in octanol/PBS mixtures, following the published literature reports (Li et al., 2019) and presented as log *D*_*7.4*_ values. Briefly, a small volume of [^18^F]**3** was added into an octanol/water mixture, and then, a series of diluted octanol/PBS mixtures was obtained, as described in the literature. The CPM in octanol and the PBS layer was obtained in a Wizard 2470 gamma counter (PerkinElmer) and was used to calculate the lipid/PBS partition coefficient. Several continuous and stable partition coefficient values were selected to calculate the log *D*_*7.4*_ values.

The *in vitro* stability of [^18^F]**3** was performed in PBS saline, 20% EtOH solution, and rat serum. In general, a solution of [^18^F]**3** (50 µL, approximately 20 KBq) was added to the PBS saline (2 mL), 20% EtOH solution (2 mL), and rat serum (2 mL), and these solutions were suspended in a water bath under 37°C for 6 h. Samples from PBS saline and 20% EtOH solution were loaded into the analytical radio-HPLC for the detection of RCP at designated time points (0, 1, 2, and 4 h), with the same condition described previously. The samples from the rat serum were added the same volume of acetonitrile to deposit the plasma protein, and the supernatant obtained by centrifugation (×12,000 r/min, 5 min) was analyzed at corresponding time points.

### 5.5 Cell uptake studies

The human FAP-expressing cell line was prepared in A549 cells (A549-FAPI), as described by Tang *et al*, and was used in this section (Western blot analysis confirmed the FAP expression, see SI), together with U87MG cell lines, which was proved to be FAP-positive (Ma et al., 2021; Hu et al., 2022; Wang et al., 2022). Briefly, all cells were divided into the following two groups: A: blocking group and B: vehicle group. All cells were cultured, grouped, and plated (approximately 2*10^6^ cells per well) before use. Cells were treated with [^18^F]**3** (74 KBq) and incubated at 37°C under a 5% CO_2_ atmosphere. Cells from the blocking group were added to the reference standard (Compound 1) in each well to reach the final concentration of 10 µM 1 h before the addition of [^18^F]**3** to investigate selective binding. Incubated at 37°C for 1 h, the medium from each well was removed, and the cells were washed twice with 1 mL of PBS. The medium and the PBS from each well were combined and counted in a Gamma counter (PerkinElmer Wizard 2470) for radioactivity counts. The cells were then handled with 1 mL of 1 M NaOH, and the lysates were also counted in the Wizard 2470 gamma counter. The uptake ratios for each group were then calculated with this equation: %Dose = (medium counts + PBS counts) * 100%/(medium counts + PBS counts + cell lysate counts).

### 5.6 Biodistribution studies

Normal Kunming mice (4 weeks, 20–25 g) were injected intravenously with about 740 KBq of [^18^F]**3** in 100 µL of PBS saline solution *via* the tail vein. All subjects were grouped by time points (n = 5). At designated time points, all subjects were sacrificed, and the organs and tissues were harvested, weighted, and counted in a PerkinElmer Wizard 2470 gamma counter. All the counts obtained from the gamma counter were decay-corrected and used to calculate the tissue biodistribution data, which is presented as the percentage of injected dose per gram of organ (%ID/g).

### 5.7 Tumor model and PET imaging study

BALB/c nude mice used in this study were obtained from the Animal Center of Sichuan University. Tumor-bearing mouse models were established by the subcutaneous injection of 100 µL of PBS suspension containing approximately 1*10^6^ tumor cells to the body side of the subjects. The tumor volume was monitored every day, and the tumor-bearing mice were ready for imaging once the tumor volume reached 0.5 mm^3^ (tumor volume can be calculated by this equation: Volume = 0.5 * long tail * short tail *short tail). Micro-PET/CT imaging studies were performed on the IRIS Micro-PET/CT system (Inviscan SAS, France). Tumor-bearing mice were injected with approximately 11.2–14.8 MBq of [^18^F]**3** (200 µL) *via* the tail vein under anaesthetization by isoflurane. Static PET images were obtained at designated time points and were reconstructed with a 3D-OSEM algorithm using a Monte Carlo-based accurate detector model. Then, 170 sec-CT acquisition was conducted with 50 kV, 1 mA X-ray output for attenuation correction and anatomical orientation. ROIs (regions of interest) were drawn on the lung, kidney, liver, and tumor by OsiriX software. SUV (standardized uptake value) of the ROIs was directly obtained from OsiriX for each subject according to this formula: SUV = Voxal value (Bq/mL) * Body weight (kg)/(Decay corrected Dose (Bq) * 1,000 (g/kg)).

## Data Availability

The original contributions presented in the study are included in the article/Supplementary Material; further inquiries can be directed to the corresponding authors.
