# Supplementary material for: Synthesis, radiolabeling, and evaluation of a (4-quinolinoyl)glycyl-2-cyanopyrrolidine analogue for fibroblast activation protein (FAP) PET imaging
Source: Front Bioeng Biotechnol. 2023 Mar 28;11:1167329. doi: 10.3389/fbioe.2023.1167329 (PMC10086185; doi:10.3389/fbioe.2023.1167329)
Supplement: Supplementary file 3 [file DataSheet1.DOCX]

Supplementary Materials for Synthesis, radiolabeling and evaluation of a (4-quinolinoyl)glycyl-2-cyanopyrrolidine analogue for Fibroblast Activation Protein (FAP) PET imaging

Ni Zhang^1, 2*^, Fei Pan^1*^, Lili Pan^1^, Wei Diao^1^, Feijing Su^3^, Rui Huang^4^, Bo Yang^5, 6^, YunChun Li^1^, Zhongzhi Qi^1#^, Wenjie Zhang^1#^ and Xiaoai Wu^1^

**Table of Contents**

[1. General information 3](#_Toc127444323)

[2. Synthesis and characterization data of compound **5**. 3](#_Toc127444324)

[3. Synthesis and characterization data of compound **6**. 4](#_Toc127444325)

[4. Synthesis of compound **7**. 5](#_Toc127444326)

[5. Synthesis and characterization data of compound **3**. 5](#_Toc127444327)

[6. Synthesis and characterization data of compound **8**. 8](#_Toc127444328)

[7. Synthesis and characterization data of compound **9**. 10](#_Toc127444329)

[8. Validation of A549-FAP cells. 12](#_Toc127444330)

[9. Tumor model and PET imaging study 12](#_Toc127444331)

1. General information

All regents, compounds and solvents were obtained from commercial suppliers, and used without further processing unless indicated. Thin layer chromatography was used to monitor organic reactions and flash column chromatography was used to purify the products. ^1^H NMR and ^13^C NMR spectra were performed on a spectrometer (Bruker AV-400, 400 MHz) in the Analysis and Testing Center of Sichuan university, and coupling constants are indicated as J with Hz (hertz), multiplicities are expressed as s, d, t, q and m for singlet, doublet, triplet, quartet and multiplet, respectively. High resolution mass spectrometry (HRMS) was carried out in a Q-TOF Premier mass spectrometer (Waters) in State Key Laboratory of Biotherapy, Sichuan University.

All protocols related to animals in this investigation were performed with the approval of the Animal Management Committee of Sichuan University. U87MG cells were obtained from ATCC, A549-FAP cells was obtained from BaoLu biotechnology Ltd. All animals were obtained from the Animal Center of Sichuan University.

1. Synthesis and characterization data of compound **5**.

*(S)-tert-butyl (2-(2-carbamoyl-4,4-difluoropyrrolidin-1-yl)-2-oxoethyl)carbamate* *(****5****)* : This compound has been reported and was synthesized according to reported literatures with minor modifications (ACS Medicinal Chemistry Letters  2013, 4, 5, 491-496). To a solution of **4** (3.9 g, 22.3 mmol) in DMF (50 mL) was added HOBT (3.6 g, 25.6 mmol) and EDCI (5.2 g, 25.6 mmol), and the mixture was stirred at room temperature for 30 minutes. Then (2S)-4,4-difluoropyrrolidine-2-carboxamide hydrochloride (4.8 g, 25.6 mmol) and DIPEA (9.2 mL, 55.8 mmol) was added, the solution was stirred at room temperature for 15 hours. The reaction mixture was then quenched with water (100 mL) and extracted with EtOAc (60 mL × 3). The organic extracts were combined, and washed with water (100 mL × 1), brine (100 mL × 1), then dried over Na_2_SO_4_ and concentrated under reduced pressure, the residue was purified by sillica column chromatography with DCM/MeOH (90 : 10) to obtain compound **5** (4.2 g), yield 62%. ^1^H NMR (400 MHz, DMSO-*d*_6_) δ 7.39 (br, 1H), 7.15 (br, 1H), 6.90 – 6.84 (m, 1H), 4.45 (dd, *J* = 9.7, 4.2 Hz, 1H), 4.08 (q, *J* = 12.7 Hz, 1H), 3.94 (q, *J* = 12.7 Hz, 1H), 3.85 – 3.66 (m, 2H), 2.95 – 2.63 (m, 1H), 2.44 – 2.20 (m, 1H), 1.38 (s, 9H).


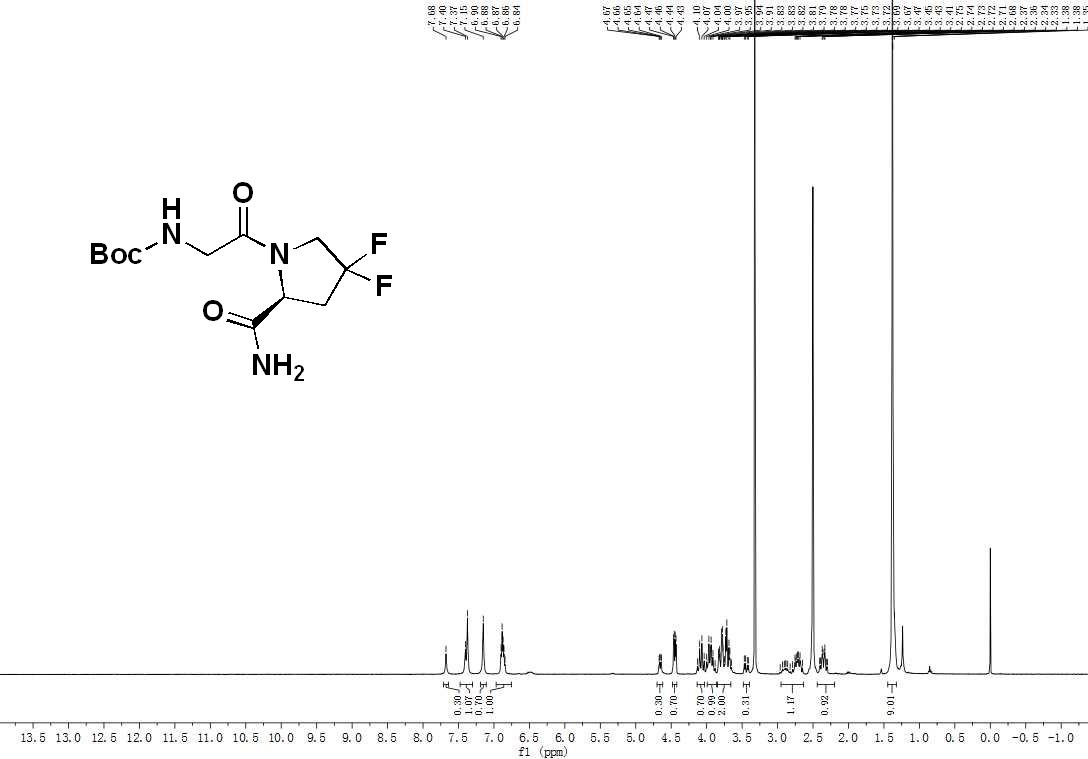


1. Synthesis and characterization data of compound **6**.

*(S)-tert-butyl (2-(2-cyano-4,4-difluoropyrrolidin-1-yl)-2-oxoethyl)carbamate (****6****)* : This compound has been reported and was synthesized according to reported literatures with minor modifications (ACS Medicinal Chemistry Letters  2013, 4, 5, 491-496). To a solution of **5** (4.1 g, 14.2 mmol) in anhydrous THF (50 mL) was added pyridine (2.8 mL, 99.5 mmol) at 0 ^o^C, and a solution of trifluoroacetic anhydride (1.1 mL, 21.3 mmol) in dichlormethan (8 mL) was added dropwise over 10 minutes. After stirring for 3 hours, the reaction mixture was concentrated under reduced pressure, then the resulting compound was redissolved in EtOAc (150 mL) and washed with water (100 mL × 1), brine (100 mL × 1), dried over Na_2_SO_4_ and concentrated under reduced pressure, the residue was purified by sillica column chromatography with DCM/MeOH (95 : 5) to obtain compound **3** (3.3 g), yield 87%. ^1^H NMR (400 MHz, Chloroform-*d*) δ 5.34 (br, 1H), 4.97 (t, *J* = 6.5 Hz, 1H), 4.06 – 3.78 (m, 4H), 2.80 – 2.71 (m, 2H), 1.46 (s, 9H).


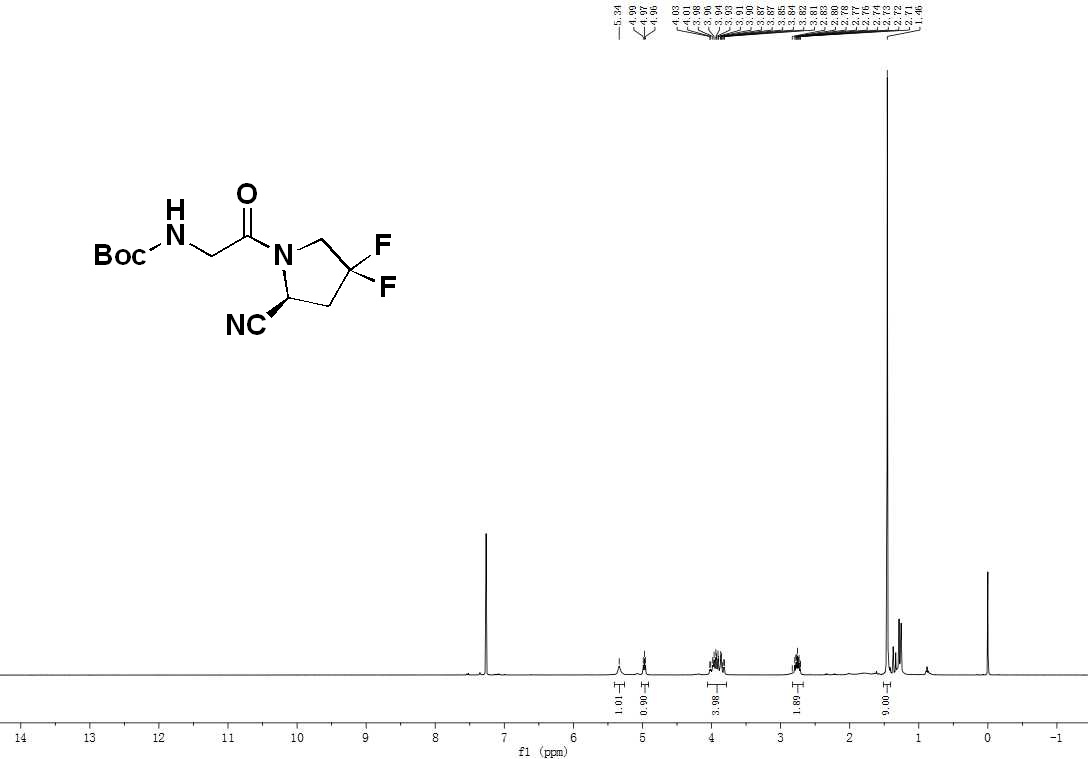


1. Synthesis of compound **7**.

*(S)-2-(2-cyano-4,4-difluoropyrrolidin-1-yl)-2-oxoethanaminium 4-methylbenzenesulfonate (****7****)* : This compound has been reported and was synthesized according to reported literatures with minor modifications (ACS Medicinal Chemistry Letters  2013, 4, 5, 491-496). To a solution of **5** (3.2 g, 11.1 mmol) in acetonitrile (60 mL) was added p-Toluenesulfonic acid (3.7 g, 16.7 mmol) , the solution was stirred at room temperature overnight. The mixture was concentrated under reduced pressure, and the residue was dissolved in diethylether (300 mL) , then the solution was cooled in an ice-water bath and kept stirring for 1 hour, during which time a lot of white solid precipitates. The crude whitish solid was collected *via* vacuum filtration, and used without further purification.

1. Synthesis and characterization data of compound **3**.

*(S)-N-(2-(2-cyano-4,4-difluoropyrrolidin-1-yl)-2-oxoethyl)-6-fluoroquinoline-4-carboxamide (3)* : This compound has been reported and was synthesized according to reported literatures with minor modifications (ACS Medicinal Chemistry Letters  2013, 4, 5, 491-496). To a solution of 6-fluoroquinoline-4-carboxylic acid (99.8 mg, 0.52 mmol) in DMF (6 mL) was added HOBT (77.8 mg, 0.58 mmol) and EDCI (110.8 mg, 0.58 mmol), and the mixture was stirred at room temperature for 30 minutes. Then compound **5** (209.4 mg, 0.58 mmol) and DIPEA (0.22 mL, 1.3 mmol) was added, the solution was stirred at room temperature for 15 hours. The reaction mixture was then quenched with water (15 mL) and extracted with EtOAc (25 mL × 3). The organic extracts were combined, and washed with water (50 mL × 1), brine (50 mL × 1), then dried over Na_2_SO_4_ and concentrated under reduced pressure, the residue was purified by sillica column chromatography with DCM/MeOH (90 : 10) to obtain compound **3** (120 mg), yield 63%. ^1^H NMR (400 MHz, Chloroform-*d*) δ 8.91 (d, *J* = 4.3 Hz, 0.2H), 8.87 (d, *J* = 4.3 Hz, 0.8H), 8.12 (dd, *J* = 9.2, 5.6 Hz, 1H), 8.00 (dd, *J* = 10.0, 2.9 Hz, 0.2H), 7.96 (dd, *J* = 10.0, 2.9 Hz, 0.8H), 7.56 – 7.49 (m, 2H), 7.23 (d, *J* = 5.0 Hz, 0.8H), 7.09 (d, *J* = 5.0 Hz, 0.2H), 5.19 (t, *J* = 6.6 Hz, 0.2H), 4.99 (t, *J* = 6.6 Hz, 0.8H), 4.62 (dd, *J* = 17.6, 5.6 Hz, 0.2H), 4.44 (dd, *J* = 17.6, 5.6 Hz, 0.8H), 4.28 (dd, *J* = 17.6, 4.2 Hz, 0.2H), 4.17 (dd, *J* = 17.6, 4.2 Hz, 0.8H), 4.16 – 3.93 (m, 2H), 2.92 – 2.89 (m, 0.4H), 2.87 – 2.77 (m, 1.6H). ^13^C NMR (101 MHz, Chloroform-*d*) δ 167.37, 167.12, 162.43 & 159.95 (d, *J* = 250.5 Hz, 1C, C-F coupling), 149.00, 145.92, 132.51, 132.42, 125.25, 120.65, 119.41, 116.02, 109.12, 52.07 (t, *J* = 31.3 Hz, 1C), 44.35, 42.27, 37.34 (t, *J* = 25.4 Hz, 1C), 28.56. HRMS ESI-MS-q-TOF for C_17_H_13_F_3_N_4_O_2_ [M+Na ]^+^ found, 385.0883 m/z; calcd mass, 385.0887.


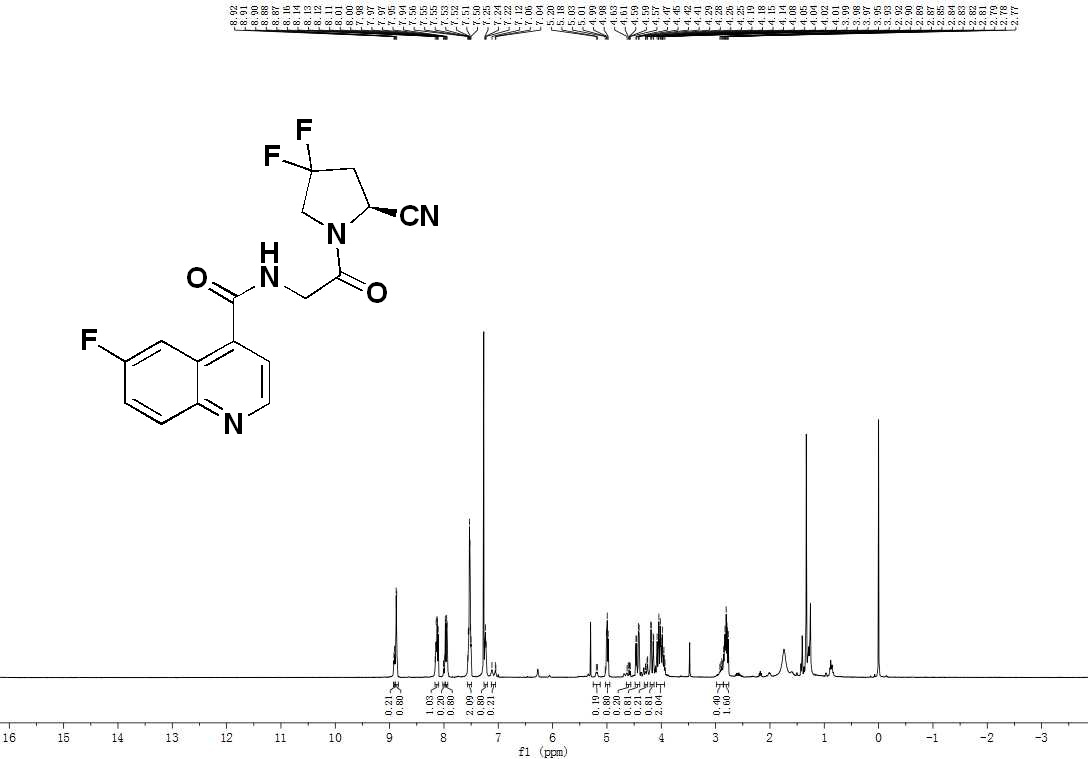


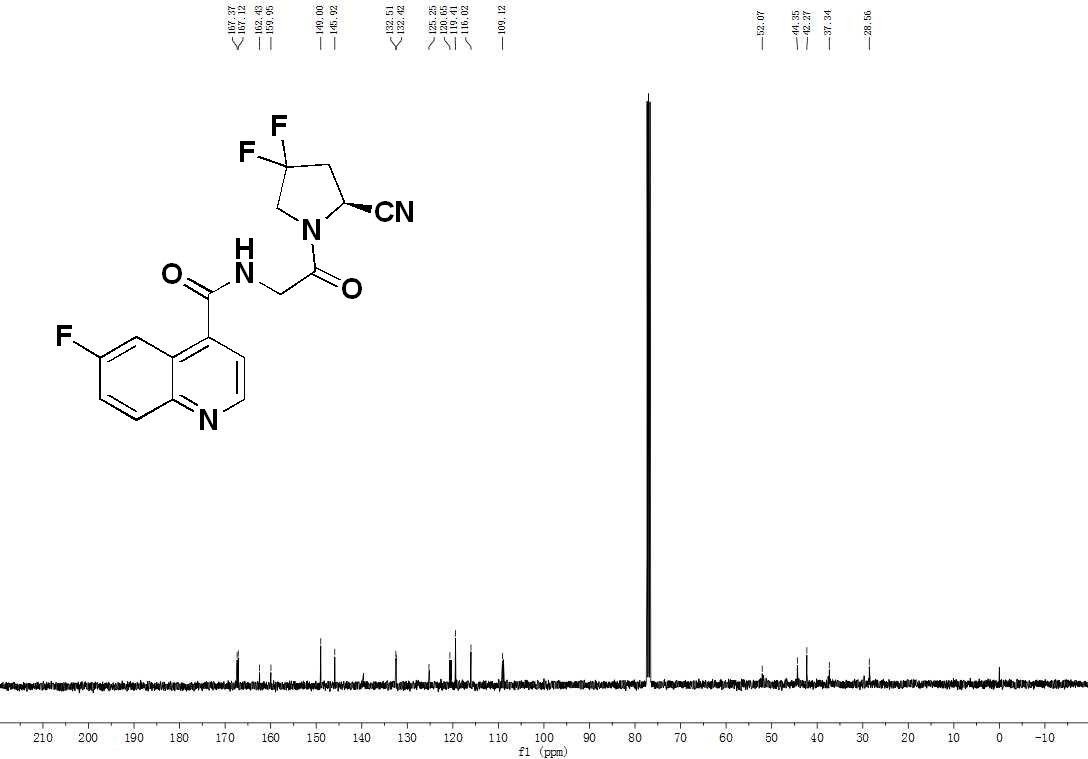

1. Synthesis and characterization data of compound **8**.

*(S)-6-bromo-N-(2-(2-cyano-4,4-difluoropyrrolidin-1-yl)-2-oxoethyl)quinoline-4-carboxamide (****8****)* : To a solution of 6-bromoquinoline-4-carboxylic acid (131.5 mg, 0.52 mmol) in DMF (6 mL) was added HOBT (77.8 mg, 0.58 mmol) and EDCI (110.8 mg, 0.58 mmol), and the mixture was stirred at room temperature for 30 minutes. Then compound **7** (209.4 mg, 0.58 mmol) and DIPEA (0.22 mL, 1.3 mmol) was added, the solution was stirred at room temperature for 15 hours. The reaction mixture was then quenched with water (15 mL) and extracted with EtOAc (25 mL × 3). The organic extracts were combined, and washed with water (50 mL × 1), brine (50 mL × 1), then dried over Na_2_SO_4_ and concentrated under reduced pressure, the residue was purified by sillica column chromatography with DCM/MeOH (90 : 10) to obtain compound **8** (152 mg), yield 69%. ^1^H NMR (400 MHz, Chloroform-*d*) δ 8.91 (d, *J* = 4.5 Hz, 0.1H), 8.87 (d, *J* = 4.5 Hz, 0.9H), 8.43 (d, *J* = 2.2 Hz, 1H), 7.98 (d, *J* = 8.9 Hz, 0.1H), 7.95 (d, *J* = 8.9 Hz, 0.9H), 7.80 (dd, *J* = 9.0, 2.2 Hz, 1H), 7.48 (d, *J* = 4.3 Hz, 1H), 7.34 (t, *J* = 5.0 Hz, 0.9H), 7.20 (t, *J* = 5.0 Hz, 0.1H), 5.19 (dd, *J* = 7.9, 5.3 Hz, 0.1H), 4.98 (dd, *J* = 7.9, 5.3 Hz, 0.9H), 4.64 (dd, *J* = 17.5, 5.8 Hz, 0.1H), 4.44 (dd, *J* = 17.5, 5.8 Hz, 0.9H), 4.27 (dd, *J* = 17.5, 4.2 Hz, 0.1H), 4.14 (dd, *J* = 17.5, 4.2 Hz, 0.9H), 4.34 – 4.05 (m, 2H), 2.82 – 2.80 (m, 0.2H), 2.79 – 2.76 (m, 1.8H). ^13^C NMR (101 MHz, Chloroform-*d*) δ 167.45, 166.96, 150.04, 147.19, 139.31, 133.65, 131.50, 127.52, 125.32, 122.25, 119.42, 116.12, 52.04 (t, *J* = 31.3 Hz, 1C), 44.36, 42.27, 37.54 (t, *J* = 25.2 Hz, 1C), 29.70.


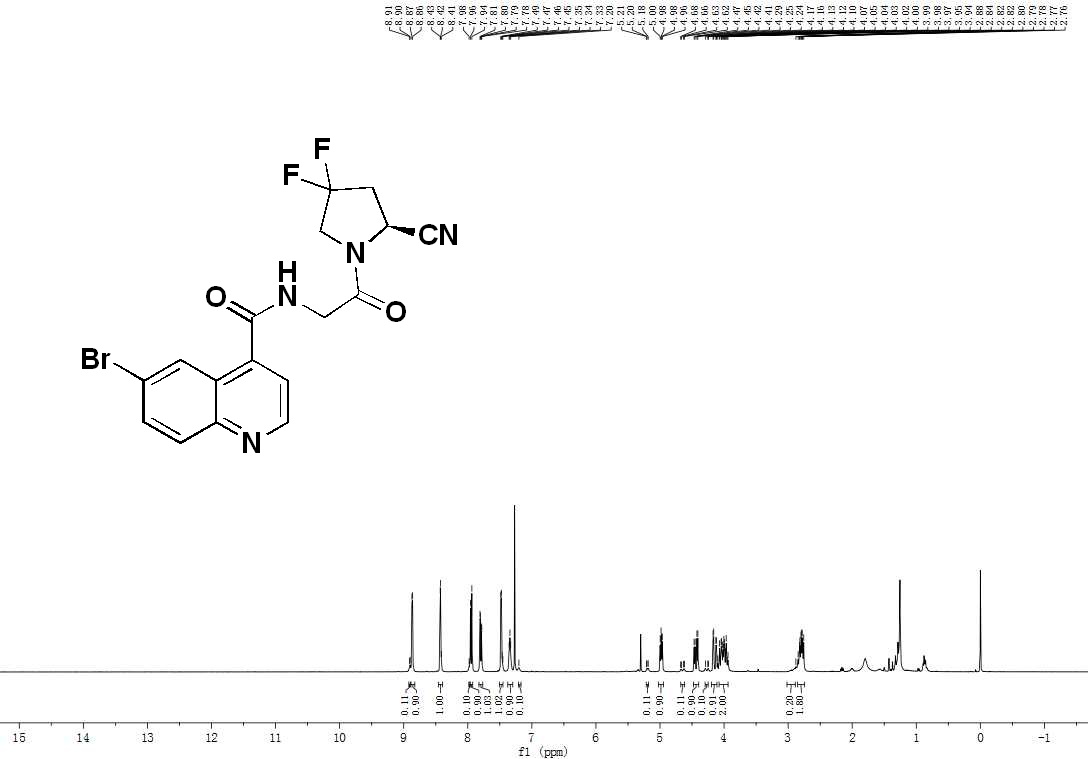


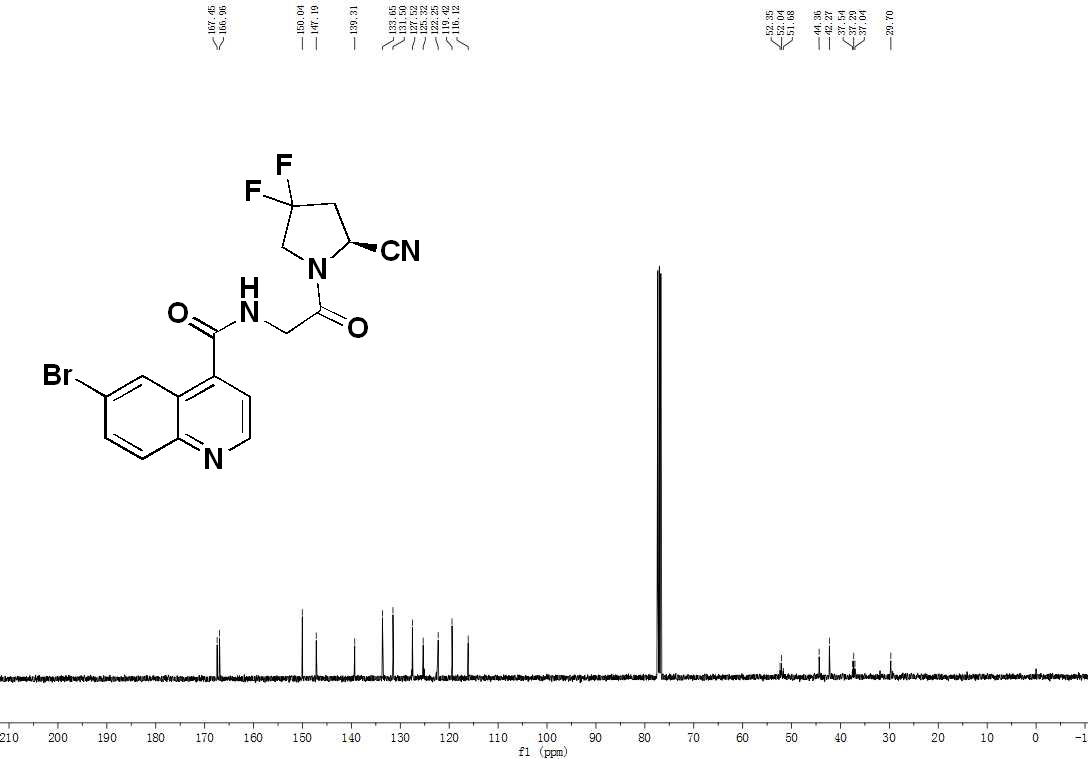


1. Synthesis and characterization data of compound **9**.

*(S)-N-(2-(2-cyano-4,4-difluoropyrrolidin-1-yl)-2-oxoethyl)-6-(4,4,5,5-tetramethyl-1,3,2-dioxaborolan-2-yl)quinoline-4-carboxamide (****9****)*: To a solution of compound **8** (120.2 mg, 0.28 mmol) in DMF (3 mL) was added bis(pinacolato)diboron (106.7 mg, 0.42 mmol), [1,1’-Bis(diphenylphosphino)ferrocene]dichloropalladium(II) (20.5 mg, 0.03 mmol) and potassium acetate (137.2 mg, 1.4 mmol) under argon. Then the solution was heated to 80 ℃ for overnight. The reaction mixture was then quenched with water (10 mL) and extracted with EtOAc (20 mL × 3). The organic extracts were combined, and washed with water (30 mL × 1), brine (30 mL × 1), then dried over Na_2_SO_4_ and concentrated under reduced pressure, the residue was purified by sillica column chromatography with DCM/MeOH (90 : 10) to obtain compound **9** (70 g), yield 52%. ^1^H NMR (400 MHz, Acetonitrile-*d*_3_) δ 8.98 (d, *J* = 4.4 Hz, 1H), 8.66 (s, 1H), 8.12 – 7.99 (m, 2H), 7.59 (d, *J* = 4.3 Hz, 1H), 7.44 (br, 1H), 5.23 (dd, *J* = 9.2, 3.1 Hz, 0.1H), 5.02 (dd, *J* = 9.2, 3.1 Hz, 0.9H), 4.47 (dd, *J* = 17.3, 6.0 Hz, 0.1H), 4.29 (dd, *J* = 17.3, 6.0 Hz, 0.9H), 4.23 – 3.93 (m, 3H), 2.90 – 2.88 (m, 0.2H), 2.88 – 2.73 (m, 1.8H), 1.37 (s, 12H).^13^C NMR (101 MHz, Acetonitrile-*d*_3_) δ 168.58, 168.35, 152.30, 150.82, 142.66, 135.02, 134.20, 129.83, 127.55, 124.75, 120.27, 118.53, 85.29 (2C), 52.68 (t, *J* = 31.3 Hz, 1C), 45.41, 42.69, 37.81 (t, *J* = 25.2 Hz, 1C), 25.20 (4C). HRMS ESI-MS-q-TOF for C_23_H_25_BF_2_N_4_O_4_ [M+Na ]^+^ found, 493.1829 m/z; calcd mass, 493.1835.


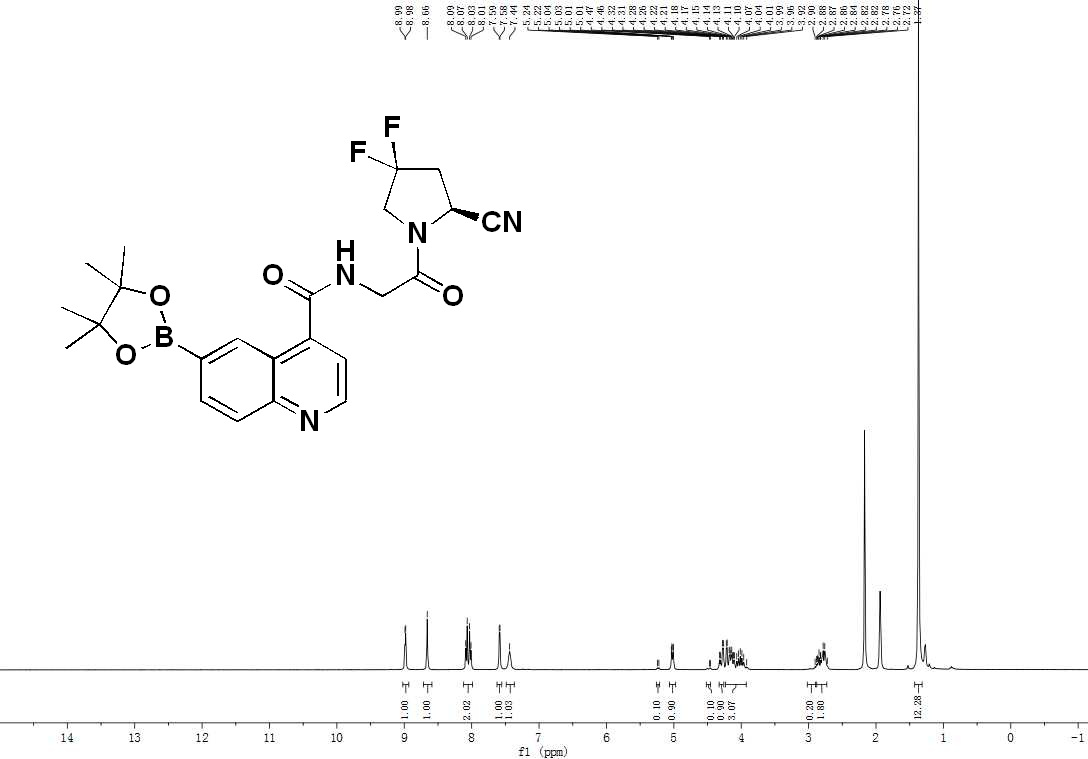


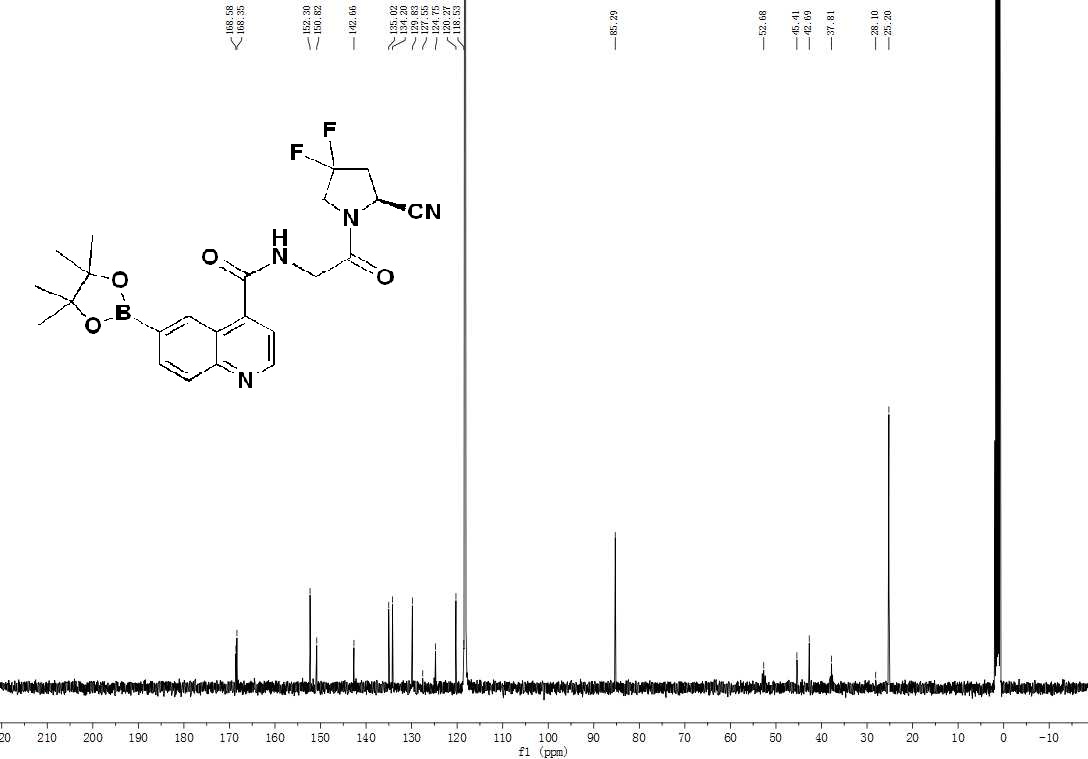

1. Validation of A549-FAP cells.

The expression of FAP-α in A549-FAP cells was confirmed by Western-Blot analysis, as showed in **Figure S1**.


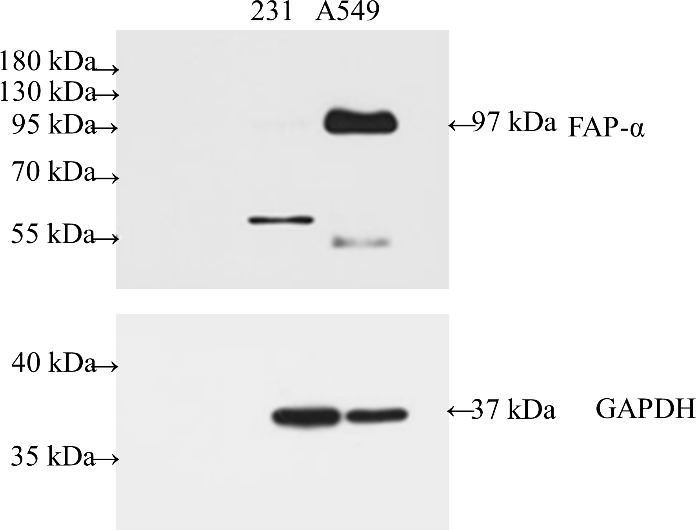


**Figure S1.** Western-Blot analysis of A549-FAP cells and MD-MBA-231 cells (negative control).

1. Tumor model and PET imaging study

BALB/c nude mice used in this study were obtained from the Animal Center of Sichuan university. Tumor-bearing mice model were established by the subcutaneous injection of 100 µL of PBS suspension containing approximately 1*10^6^ tumor cells to the body side of the subjects. The tumor volume was monitored every day and the tumor-bearing mice were ready for imaging once the tumor volume reach 0.5 mm^3^ (tumor volume can be calculated by this equation: Volume = 0.5 * long tail * short tail *short tail). Micro-PET/CT imaging studies were performed on the IRIS Micro-PET/CT system (Inviscan SAS, France). Tumor-bearing mice were injected with approximately 11.2-14.8 MBq of [^18^F]**3** (200 µL) via tail vein under anaesthetization by isoflurane. Static PET images were obtained at designated time points and were reconstructed with a 3D-OSEM algorithm using a Monte-Carlo based accurate detector model. A 170 sec-CT acquisition was conducted with 50 kV, 1 mA X-ray output for attenuation correction and anatomical orientation. ROIs (regions of interest) were drawn on lung, kidney, liver and tumor by Osirix software. SUV (standardized uptake value) of the ROIs was directly obtained from Osirix for each subject according to this formula: SUV = Voxal value (Bq/mL) * Body weight (kg)/(Decay corrected Dose (Bq) * 1000 (g/kg)).
